# Long noncoding RNA LINC00641 promotes renal cell carcinoma progression via sponging microRNA-340-5p

**DOI:** 10.1186/s12935-021-01895-y

**Published:** 2021-04-14

**Authors:** Jianping Zhang, Shengming Jin, Wenjun Xiao, Xuchao Zhu, Chengyou Jia, Zongming Lin

**Affiliations:** 1 Department of Urology, Zhongshan Hospital, Fudan University, Shanghai, 200032 China; 2 Department of Urology, Fudan University Shanghai Cancer Center, Shanghai, China; 3 Department of Oncology, Shanghai Medical College, Fudan University, Shanghai, China; 4 Department of Nuclear Medicine, Shanghai Tenth People’s Hospital, Tongji University School of Medicine, Shanghai, China

**Keywords:** Renal cell carcinoma, LINC00641, Long non-coding RNA, MiR-340-5p, MicroRNA, Cancer progression

## Abstract

**Background:**

Emerging evidences have revealed that long non-coding RNAs (lncRNAs) have played critical roles in tumor occurrence and progression. LINC00641 has been reported to be involved in the initiation and development of several cancers in the recent years. However, the detailed biological role of LINC00641 in renal cell carcinoma (RCC) remains largely unclear.

**Methods:**

In this study, the expression and biological function of LINC00641 were assessed in renal carcinoma both in vitro and in vivo. Cell proliferation, migration and colony formation assay were performed to explore the effect of LINC00641on growth, progression and invasion of RCC cell. qRT-PCR, flow cytometry and luciferase reporter assay and in vivo tumorigenicity assay were also carried out.

**Results:**

The expression of LINC00641 was overexpressed in RCC tissues and cell lines, and high LINC00641 expression was correlated with tumor-node-metastasis stage. Furthermore, Silencing of LINC00641 remarkably inhibited the ability of cell proliferation, colony formation, and invasive capacities, as well as increasing the apoptotic rates of RCC cells in vitro. Mechanistically, miR-340-5p was validated to be targeted by LINC00641 and knockdown of miR-340-5p counteracted LINC00641 silencing-mediated inhibition of RCC progression. In addition, in vivo experiment confirmed the findings discovered in vitro.

**Conclusions:**

These results suggested that LINC00641 promoted the progression of RCC by sponging miR-340-5p.

## Introduction

Renal cell carcinoma (RCC) represents one of the ten most common cancers in men and is characterized by high incidence and high mortality rates all over the world [1, 2]. Despite advances in the treatment of RCC have been made over the past decade, ~ 40% of patients with RCC would die due to local or distant metastasis at the time of diagnosis and the resistance to chemotherapy [1, 3]. Increasing studies have demonstrated the underlying mechanisms of RCC pathogenesis to develop more effective therapeutic strategies, however, the molecular mechanisms underlying RCC initiation and progression remain largely unknown[4–6]. Thus, a better understanding of the molecular mechanisms driving RCC progression would identify promising diagnostic markers and therapeutic targets to improve patients with RCC.

Long noncoding RNAs (lncRNAs), known as RNAs longer than 200 nucleotides with limited or no protein-coding capacity, played multiple biological functions in pathologic process at both transcriptional and post-transcriptional level [7–9]. Increasing evidence indicated that lncRNAs were dysregulated in many human cancers and involved in carcinogenesis as oncogenes or tumor suppressors, including RCC [10–14]. HOTAIR, lncARSR, LUCAT1, and MALAT1 have been reported to regulate RCC cell growth, apoptosis, migration and/or invasion, and Sunitinib resistance [14–17], suggesting lncRNAs dysfunction might be associated with RCC initiation and participated in RCC progress. Although various lncRNAs have been discovered, more studies are required to explore the roles of lncRNAs during RCC progression. LncRNA LINC00641 is located in chromosome 14q11.2 and usually low expression in normal state. Recent reports have revealed that the expression of LINC00641 was increased and involved in the tumorigenicity of bladder cancer, non-small-cell lung cancer (NSCLC) and acute myeloid leukemia (AML) [18–20]. However, the function and underlying molecular mechanisms of LINC00641 in RCC have not been reported.

In the present study, the expression of LINC00641 was detected in RCC tissues and cells. Loss-of-function experiments suggested that LINC00641 silencing suppressed RCC proliferation and invasion. Furthermore, the underlying mechanism of the interaction between PCAT1 and miR-326 was identified.

## Materials and methods

### Patients and sample collection

Fresh RRC tissues and paired adjacent tissues were obtained from patients undergoing surgical procedures at the Department of Urology of Zhongshan Hospital Affiliated Fudan College between March 2010 and December 2013 with permit number: 20190617AF. The inclusion criteria are as follows: (1) undergone radical or partial nephrectomy at our center; (2) pathologically diagnosed ccRCC; (3) adults with age ≥ 18-year-old; (4) None of any radiotherapy or chemotherapy was received prior to surgical treatment. The exclusion criteria are: (1) accompanied with other malignant tumors; (2) preoperative systemic treatment; (3) without complete/available follow-up/clinical/pathological data. Written informed consents were acquired from all enrolled patients prior to the study. This study was conducted in accordance with the Ethic Committee of Zhongshan Hospital Affiliated Fudan College and based on the ethical principles for medical research involving human subjects of the Helsinki Declaration. All samples were immediately snap-frozen in liquid nitrogen and stored at − 80 °C until use.

### RNA extraction and qRT-PCR

Total RNA was extracted from tissue samples and cell lines using TRIzol Reagent (Life Technologies, CA, USA). RNA was reverse transcribed into complementary DNA (cDNA) using Primescript RT reagent kit (Takara, Dalian, China) or a commercial miRNA reverse transcription PCR kit (GenePharma, China) according to the manufacturer’s protocols. QRT-PCR was performed using SYBR Green (Takara, Dalian, China) using Applied Biosystems 7500 instrument. Primer sequences used in this study are listed as follow: LINC00641 forward: 5′-CACTTTTGCAGACCCTCACA-3′, reverse: 5′-ACTTGACGGGTGGATTCTTG-3′; GAPDH forward: 5′-GAAGGTGAAGGTCGGAGTC-3′, reverse: 5′-GAAGATGGTGATGGGATTTC-3′; U6 forward 5′-CTCGCTTCGGCAGCACA-3′ and reverse 5′-AACGCTTCACGAATTTGCGT-3′. U6 and GAPDH were taken as the internal control.

### Cell culture

Human renal cell carcinoma cells (GRC-1, 786-O, SN12-PM6, A498 and ACHN) and normal kidney cell (HK-2) were purchased from the Shanghai Institute of Biological Sciences (Shanghai, China) and maintained in RPMI 1640 medium supplemented with 10% fetal bovine serum (Life Technologies) 1% penicillin/streptomycin at 37 °C in a humidified incubator.

### Plasmids construction and virus packaging

shRNAs specially targeting LINC00641 (shRNA) were synthesized by RiboBio (RiboBio, Guangzhou, China) and cloned into the pLKO.1-TRC cloning vector (AddGene, MA, USA). Scrambled sequence was generated as negative control (SCR). Lentivirus was produced by transfection of plasmids into 293 T cells with Lipofectamine 2000 (Life Technology) following the manufacture’s instruction.

### Cell proliferation assays

A total of 2 × 10^3^ renal cell carcinoma cells were seeded into each well of 96-well plates and incubated at 37 °C in a 5% CO_2_ humidified incubator. 10 µL of CCK-8 reagent (Dojindo Laboratories, Kumamoto, Japan) was added to each well at 24, 48, 72 and 96 h. After 2 h incubation, the optical density was measured at 450 nm in a microplate reader (Bio-Rad, CA, USA).

### Colony formation assay

A total of 800 cells were seeded into 6-well plates, and every condition was evaluated in triplicate. The medium was replaced every 3 days. After 2 weeks, the cell colonies were fixed with 4% paraformaldehyde for 30 min and stained with 0.5% crystal violet for 30 min. The colonies were defined as > 50 cells/colony and cell colonies were photographed and counted.

### Flow cytometry analysis assay

Cells in the logarithmic growth phase were collected and stained with Annexin V-FITC and PI according to the manufacturer’s instructions (BD Biosciences, NJ, USA). Then cell apoptosis was determined using flow cytometry (Beckman, CA, USA).

### Migration assay

A total of 1 × 10^4^ cells in 100 μL serum-free medium were seeded into the upper Boyden chamber (8-μm pores; Corning, NY, USA) coated without or with Matrigel (BD Biosciences). Culture medium supplemented with 10% fetal bovine serum was added to the lower chamber as a chemoattractant. After incubation for 24 h, the migrated or invaded cells were fixed, stained with 0.5% crystal violet. The cells were counted in 10 random fields using an inverted microscope (magnification × 200).

### Luciferase reporter assay

The sequences of LINC00641 containing miR-340-5p binding site or mutant binding site into pmirGLO vector (Promega, MI, USA). The luciferase reporter vectors and miR-340-5p mimics were co-transfected into A498 and ACHN cells by Lipofectamine 2000 reagent and luciferase activity was determined with the Dual-Luciferase Reporter Assay System (Promega) following the manufacturer’s instructions.

### Western blotting analysis

For Western blotting, proteins were extracted using 1 × RIPA buffer (Cell Signaling Technology, MA, USA) containing protease inhibitor cocktail (Roche, Switzerland) for 30 min on ice. A total of 60 mg of protein were separated on 8–10% SDS-PAGE gels and then transferred to a PVDF membrane (Millipore, MA, USA). The membranes were blocked in 5% non-fat milk and washed with 1 × TBST. Next, the membrane was incubated with the appropriate primary antibody as follows: anti-c-Myc (Cell Signaling Technology), anti-MMP-2 (Cell Signaling Technology), anti-CyclinD1 (Cell Signaling Technology), or anti-β-Actin (Cell Signaling Technology), which was used as a loading control. Finally, target protein bands were visualized with an ECL Substrate Kit (Thermo Fisher Scientific, USA).

### In vivo tumorigenicity assay

4-weeks old female athymic nude mice (BALB/c Nude) were purchased from Shanghai SLAC Laboratory Animals Co., Ltd. (Shanghai, China). For the tumorigenicity assay, 1 × 10^7^ cells ACHN transduced with SCR or LINC00641 shRNA were subcutaneously inoculated into the left flank of nude mice. Tumor size was measured every week with calipers and tumor volume was calculated using the following formula: volume = (length × width^2^) × 1/2. All mice were sacrificed 4 weeks after subcutaneous inoculation, and the tumors tissues were collected for further IHC. All animal experiments were carried out in accordance with the NIH Guide for the Care and Use of Laboratory Animals with protocols approved by the Animal Care and Use Committee at Fudan University.

### Statistical analysis

Data represent the mean ± standard deviation (SD). Two-tailed Student’s t-test and Chi-squared test to evaluate differences between two groups. Correlations were analyzed by Pearson’s correlation test. Relationships between LINC00641 expression and overall survival (OS) rate were analyzed using the log-rank test and Kaplan–Meier curves. A value of P < 0.05 was considered statistically significant. All of the statistical analyses were performed using R software (version 3.5.1, http://www.r-project.org/) and SPSS (version 21.0, IBM, NY, USA).

## Results

### LINC00641 is highly expressed and associated with overall survival in RCC

**Fig. 1:**
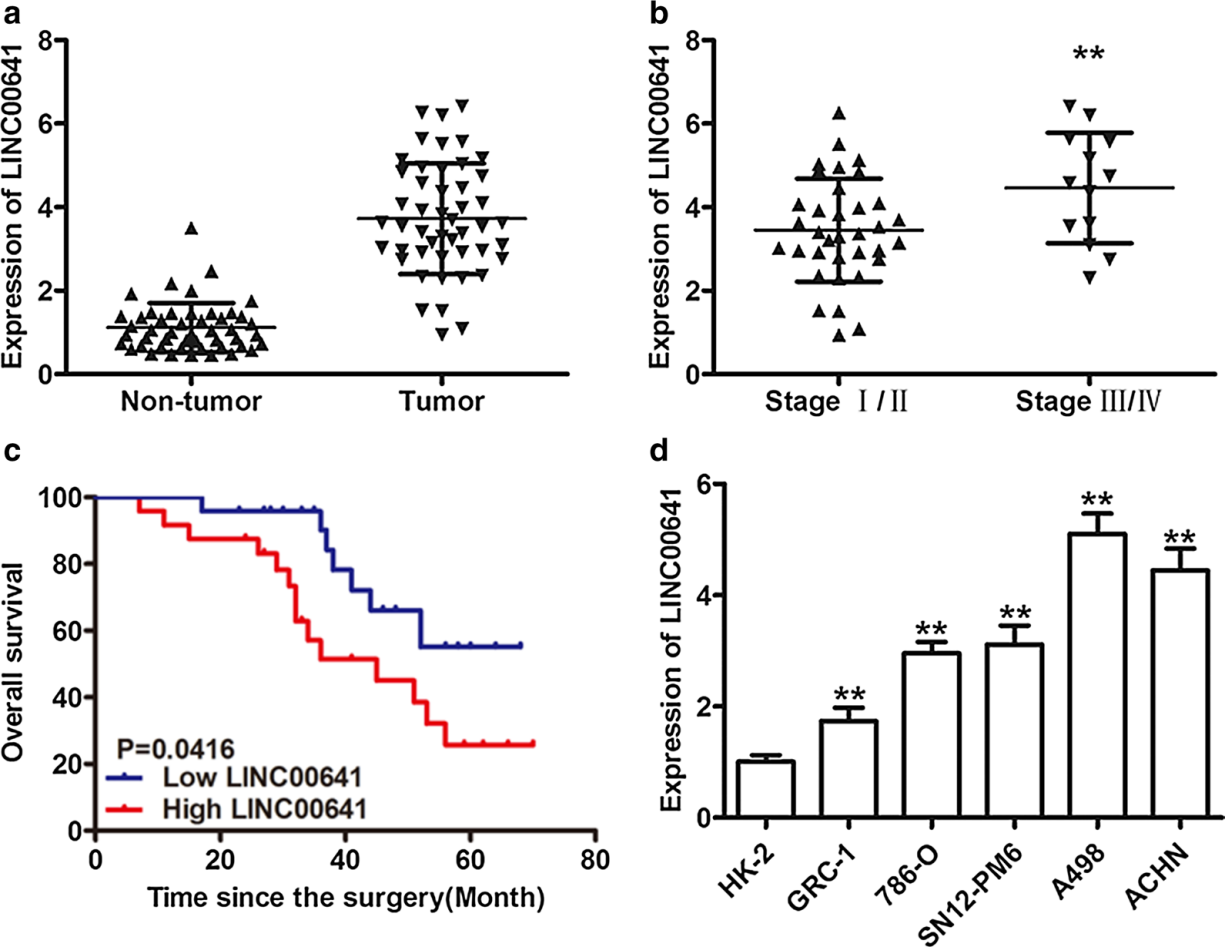
Expression of LINC00641 and its prognostic significance in RCC patients. **a** LINC00641 expression level was determined in tumor and paired adjacent non-tumor tissues by qRT-PCR. **b** Relative LINC00641 expression in RCC with different TNM stages. **c** Kaplan–Meier survival analysis according to LINC00641 expression in 48 pair RCC patients. **d** Relative LINC00641 expression in 5 RCC cell lines and human renal tubular epithelial cell line (HK-2) were detected by qRT-PCR. **P < 0.01

To study the possible role of LINC00641 in RCC, the expression levels of LINC00641 were detected in 48 cases of RCC tissue samples and the adjacent normal tissues by qRT-PCR. qRT-PCR results showed that LINC00641 was increased in human RCC tissues compared with adjacent normal tissues (Fig. 1a, P < 0.01). Then, we divided all RCC patients into high or low LINC0064 expression level groups based on the median value. Elevated LINC0064 expression was associated with advanced TNM stage (Fig. 1b, P < 0.01) and a poor prognosis (Fig. 1c, P < 0.01). The expression of LINC0064 was correlated with the patients’ clinical T stage and metastasis (Table 1). Furthermore, LINC00641 expression was significantly increased in RCC cell lines compared with the human renal tubular epithelial cell line HK-2 (Fig. 1d, P < 0.01). Taken together, these results indicate that LINC00641 is frequently upregulated in RCC.

**Table 1:** Relationship between LINC00641 expression and clinicopathological features of RCC patients

Clinicopathological features	LINC00641 expression		P value^a^
	Low	High	
Gender			0.76
Male	16	15	
Female	8	9	
Age			0.93
≤ 50	6	8	
> 50	18	16	
T stage			**0.0035**
T1/T2	22	13	
T3/T4	2	11	
N stage			0.06
N0	22	17	
N +	2	7	
Metastasis			**0.016**
No	22	15	
Yes	2	9	
Tumor size (cm)			0.38
≤ 5	13	10	
> 5	11	14	

P < 0.05 was considered statistically significant (bold values)

^a^Comparing between high and low LINC00641 expression group using chi-square test

### Knockdown of LINC00641 prohibits RCC cell growth and invasion in vitro

**Fig. 2:**
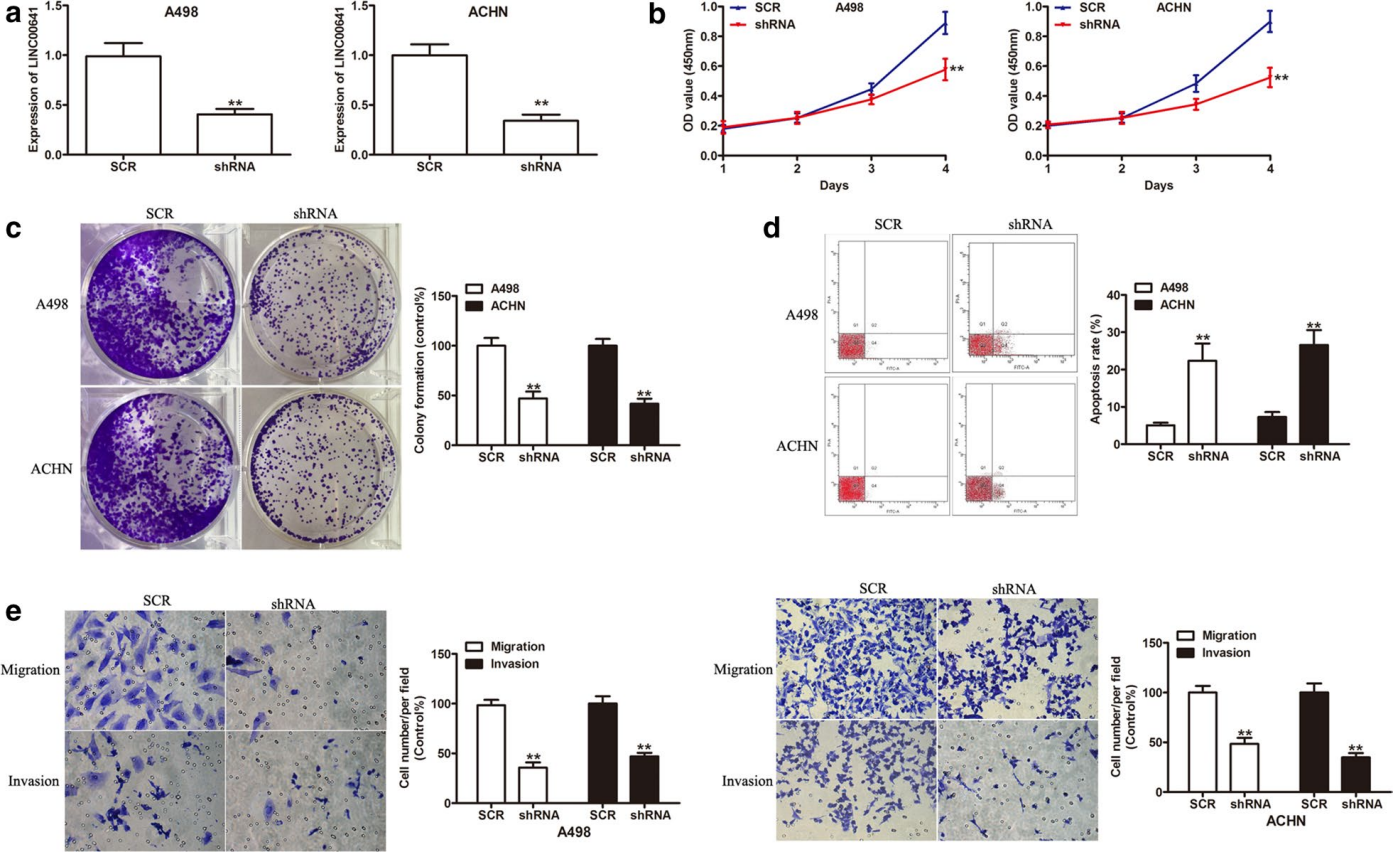
Knockdown of LINC00641 inhibits RCC cell proliferation and invasion in vitro. **a** Analysis of the LINC00641 expression level in A498 and ACHN cells expressing shLINC00641 by qRT-PCR. **b** Growth curve of RCC cells transduced with SCR or shRNA by CCK-8 assays. **c** Proliferation and **d** apoptosis of RCC cells transduced with SCR or shRNA as determined by colony formation assay or flow cytometry assay, respectively. **e** Representative photographs of migrated or invaded cells transduced with SCR or shRNA as determined by transwell migration assay and invasion assay. **P < 0.01

To explore the role of LINC00641 in RCC, two stable cell lines with LINC00641 silencing via shRNA-mediated depletion of LINC00641 were established. As shown in Fig. 2a (P < 0.01), the expression of LINC00641 was successfully knocked down in A498 and ACHN cells, which was validated by qRT-PCR. CCK-8 assays showed downregulation of LINC00641 remarkably decreased cell growth rates in both A498 and ACHN cells (Fig. 2b, P < 0.01). To further study the effect of LINC00641 on cell proliferation, clone formation assays were applied to investigate the cell proliferative abilities in LINC00641-repressing A498 and ACHN cells and the data showed that less clonogenicities was observed after LINC00641 silencing (Fig. 2c, P < 0.01). To determine the effects of LINC00641 on cell apoptosis, an obvious increase of apoptosis was observed by flow cytometry assays (Fig. 2d, P < 0.01). In addition, invasion assay results showed that the invasive capability was significantly impaired by in A498 and ACHN cells after LINC00641 silencing (Fig. 2e, P < 0.01). These data indicated that LINC00641 played an essential role in regulating RCC growth and metastasis.

### LINC00641 acted as a sponge for miR-340-5p

**Fig. 3:**
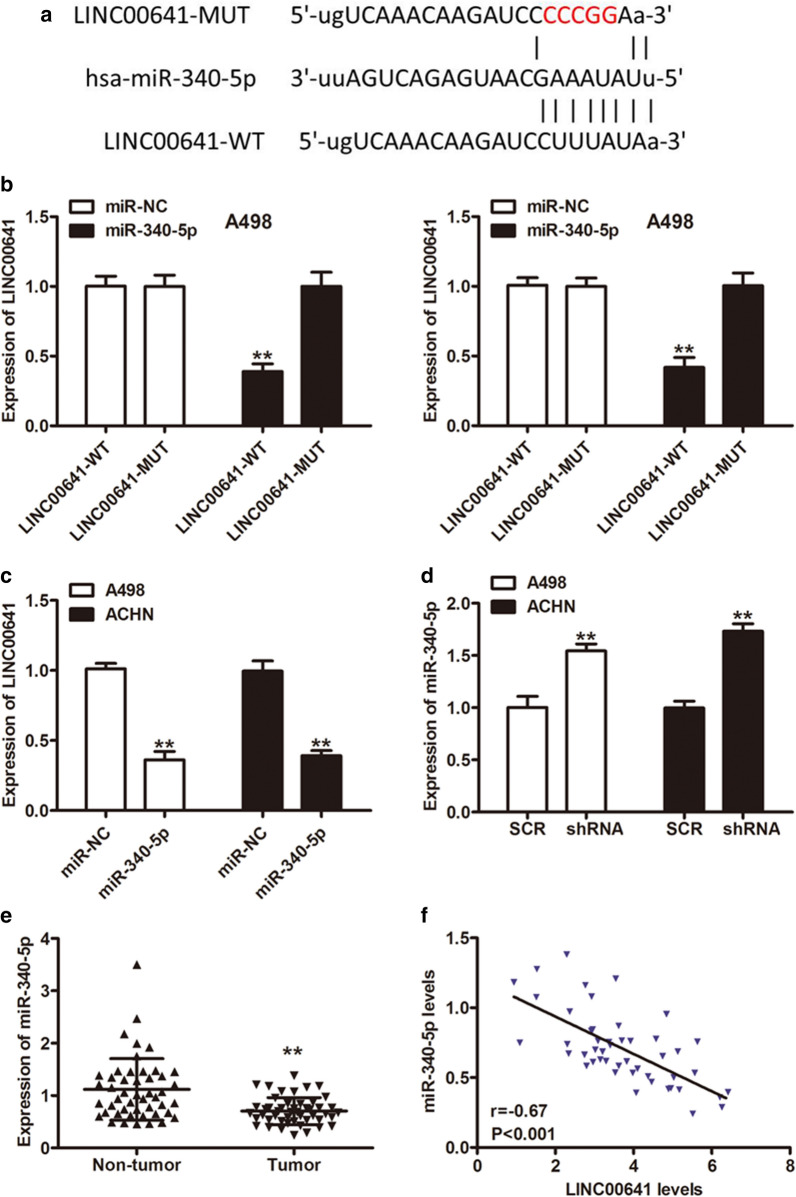
LINC00641 serves as a sponge of miR-340-5p. **a** Schematic of the predicted miR-340-5p binding site on LINC00641. **b** Luciferase activity of LINC00641-WT or LINC00641-MUT was assessed in A498 and ACHN cells transfected with miR-340-5p mimics or miR-NC. **c** Relative LINC00641 expression in A498 and ACHN cells transfected with miR-340-5p mimics or miR-NC. **d** miR-340-5p expression in A498 and ACHN cells transduced with SCR or shRNA by qRT-PCR. **e** qRT-qPCR assays were utilized to measure miR-340-5p expression in RCC tissues and paired normal tissues. **f** The inverse correlation between LINC00641 and miR-340-5p levels in RCC tissues by Pearson’s correlation. **P < 0.01

Accumulating evidence has indicated that lncRNAs can regulate target gene expression by interacting with microRNA (miRNA) as a competing endogenous RNA (ceRNA). To determine the molecular mechanism by which LINC00641 mediated RCC cell proliferation and invasion, the online database (http://starbase.sysu.edu.cn/starbase2/index.php) was used to predict the potential target miRNAs that could bind with the LINC00641 sequence and miR-340-5p was selected as the best potential target of LINC00641 (Fig. 3a). To further confirm the interaction between LINC00641 and miR-340-5p, luciferase reporter assay was performed and transfection of miR-340-5p mimics significantly decreased the relative luciferase activity of WT of LINC00641, but did not affect that of MUT both in A498 and ACHN cells (Fig. 3b, P < 0.01). LINC00641 was markedly downregulated when the RCC cells were transfected with miR-340-5p mimics (Fig. 3c, P < 0.01). Meanwhile, the expression of endogenous miR-340-5p was significantly upregulated when LINC00641 was knocked down in the RCC cells (Fig. 3d, P < 0.01). In addition, miR-340-5p was notably downregulated in RCC tissues compared with the adjacent normal tissues (Fig. 3e, P < 0.01) and a significant inverse correlation was observed between miR-340-5p and LINC00641 expression levels in RCC samples (Fig. 3f, P < 0.01). These results indicated that LINC00641 may function as a ceRNA of miR-340-5p.

### Inhibition of miR-340-5p partially blocks the effects of LINC00641 silencing on RCC cell proliferation and invasion

**Fig. 4:**
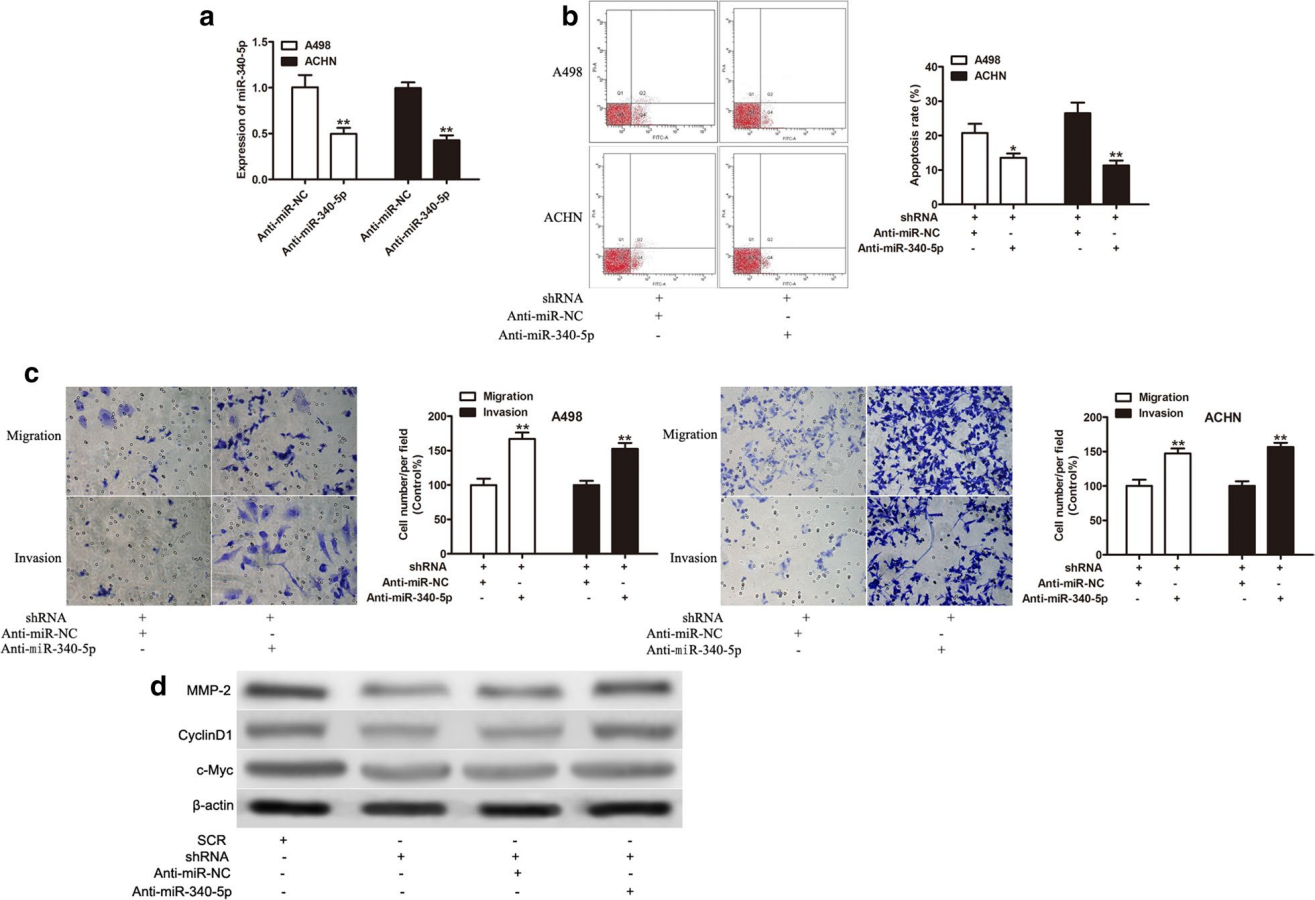
Inhibition of miR-340-5p abolished LINC00641 effects on cell apoptosis and invasion. **a** The expression of miR-340-5p was detected after transfected with anti-miR-340-5p by qRT-PCR. Cell apoptosis (**b**) and cell invasion (**c**) of RCC cells as indicated treatment. **d** The expression of c-Myc, CyclinD1 and MMP-2 protein in A498 cell lines as indicated treatment. *P < 0.05, **P < 0.01

Our above results indicated that LINC00641 silencing inhibited the cell proliferation and invasion and LINC00641 functioned as a direct target of miR-340-5p, next we explored whether LINC00641 affected the cell proliferation and invasion of RCC cells via miR-340-5p. MiR-340-5p inhibitors (Anti-miR-340-5p) were transfected to stably LINC00641 silencing RCC cells and conducted CCK-8, colony formation, and invasion accordingly. MiR-340-5p was effectively knocked down by Anti-miR-340-5p transfection in both cell lines (Fig. 4a, P < 0.05). Inhibition of miR-340-5p expression significantly abolished the inhibitory effects of LINC00641 silencing on apoptosis (Fig. 4b, P < 0.01) and invasion (Fig. 4c, P < 0.01). However, there was no significant difference in cell proliferation and colony formation (data not shown). According to the previous studies, c-Myc, CyclinD1 and MMP-2 were regulated by miR-340-5p. Then we assess whether LINC00641 could modulate the expression of c-Myc, CyclinD1 and MMP-2 in A498 cells. As we expected, LINC00641 silencing cloud effectively reduced the expression of c-Myc, CyclinD1 and MMP-2, and anti-miR-340-5p attenuated this phenomenon (Fig. 4d). Thus, these results demonstrated that LINC00641 might downregulate miR-340-5p expression to promote RCC progression.

### Inhibition of LINC00641 suppressed RCC tumourigenesis in vivo

**Fig. 5:**
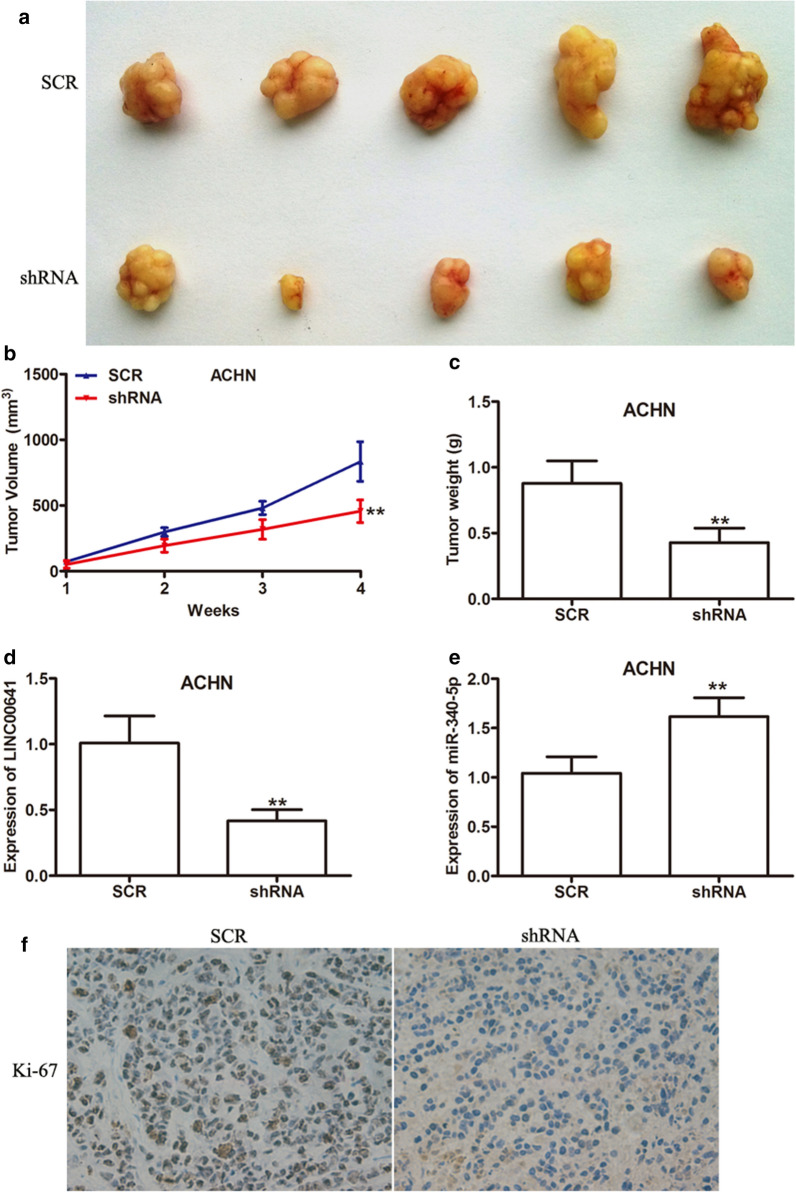
In vivo* experiments* confirmed the effects of LINC00641 silencing on tumor growth. **a** Photographs of tumors excised 4 weeks after inoculation of stably transduced with SCR or shRNA ACHN cells into nude mice. Tumor growth curve (**b**) and weight (**c**) of mouse xenografts subcutaneously injected with ACHN cells with stable LINC00641 knockdown. The expression levels of LINC00641 (**d**) and miR-340-5p (**e**) in dissected tumour tissues were detected by qRT-PCR assay. **f** Xenografts tissues were immunostained for Ki-67. **P < 0.01

To explore whether LINC00641 affects tumor growth in vivo, LINC00641 stable knockdown ACHN cells were injected into male nude mice to construct a xenograft tumor model. As shown in Fig. 5a (P < 0.01), tumors from LINC00641 stable knockdown were dramatically smaller than those formed in the SCR group. In addition, the efficient knockdown of LINC00641 significantly slowed tumor growth (Fig. 5b, P < 0.01) and decreased tumor weight (Fig. 5c, P < 0.01) compared with SCR group. Moreover, the expression of LINC00641 and miR-340-5p were detected in xenograft tumor and LINC00641 was decreased (Fig. 5d, P < 0.01), while miR-340-5p expression was increased due to LINC00641 upregulation (Fig. 5e). In addition, immunohistochemistry (IHC) staining assays revealed that LINC00641 knockdown group had lower Ki-67 protein levels (Fig. 5f, P < 0.01).

## Discussion

RCC is one of the most prevalent malignant tumors with more than 100,000 patients died annually [21]. The 5-year survival rate of patients with RCC is very poor because of its diagnosis at advanced stages or chemotherapeutic resistance [22, 23]. The rapid development of RNA genomics has revealed that lncRNAs are potential biomarkers and involved in the modulation of cancer cell behavior [7, 12, 24]. Our present study illustrated the expression level and correlation between LINC00641 and miR-340-5p during progression of RCC.

LINC00641 have been shown to serve as important regulators in several cancers, including bladder cancer, NSCLC, gastric cancer, breast cancer and acute myeloid leukemia (AML). The expression pattern of LINC00641 was highly expressed in AML specimens and cell lines and the silence of LINC00641 inhibited the proliferation, migration, invasion, and cell cycle arrest cells, while inducing their apoptosis in AML [20]. The expression of LINC00641 was also found to be high in gastric cancer. It demonstrated that LINC00641 promoted cell migration and proliferation and mediated oxaliplatin resistance by activating autophagy [25]. Consisted with the results, LINC00641 was also increased in RCC and high LINC00641 expression was associated with TNM stage and metastasis. Deletion of LINC00641 inhibited the proliferation and invasion of RCC cells as well as tumor growth in vivo. However, LINC00641 was downregulated in patients with bladder cancer and overexpression of LINC00641 markedly inhibited the proliferation, migration and invasion of bladder cancer cells [18]. In addition, LINC00641 was downregulated in patients with NSCLC and exerted tumor-suppressive role by suppressing cell proliferation and inducing cell apoptosis through sponging miR-424-5p. [19]. The inconsistency might be the tissue specificity and the expression of LINC00641 was very low in normal state (https://www.ncbi.nlm.nih.gov/gene/283624), which consisted with our results. Therefore, more studies are required to investigate the biological functions and molecular mechanisms of LINC00641 in a variety of cancers.

Many studies have suggested that lncRNA could interact with miRNA to regulate gene expression by acting as a competitive endogenous RNA of miRNA [26, 27]. Through bioinformatics analysis, LINC00641 was identified as a potential target of miR-340-5p. Studies have reported that miR-340-5p was downregulated and acted as a tumor suppressor in some types of human cancers, including glioblastoma multiforme, NSCLC, colorectal cancer, hepatocellular cancer, endometrial cancer and breast cancer [28–33]. The expression of miR-340-5p is low in glioblastoma multiforme and it played a role of inhibiting tumor growth [34]. Another study found that the expression of miR-340-5p was low in breast cancer and Overexpressed miR-340-5p inhibited cell proliferation and drug resistance to docetaxel with enhanced cell apoptosis of breast cancer cells [35]. Consisted with these results, miR-340-5p was markedly decreased in RCC tissues and an invert relation between LINC00641 and miR-340-5p. Interestingly, LINC00641 silencing remarkably reduced the proliferation, invasion, and induced the apoptosis of RCC cells, which could be abolished by anti-miR-340-5p. Taken together, these results indicated that LINC00641 promoted RCC progression via negative regulation of miR-340-5p. Likewise, another study also found that LncRNA LINC00662 promotes colon cancer tumor growth and metastasis by competitively binding with miR-340-5p, suggesting that miR-340-5p might be an important target in the mechanism of competitive endogenous RNA [31].

However, some limitations exist in this study. Firstly, the limited patient samples might not fully substantiate the accuracy of the results. Secondly, detailed investigation of genes that comprise the lncRNA LINC00641/miR-340-5p axis should also yield further insight into the mechanism by which lncRNA LINC00641 overexpression induces kidney cancer progression. And the relationship between lncRNA LINC00641 and other potential targeting miRNAs needed more attentions and researches. Thirdly, we just focused on one kidney cancer cell line, the A498 and ACHN cell line, which made our research not strict adequate and the experimental conclusion was not sufficient enough. Fourthly, the specific role of lncRNA LINC00641/miR-340-5p axis in kidney cancer remains to be further investigated.

In conclusion, the current study provided the evidence that LINC00641 exerted an oncogenic role in human RCC. The LINC00641/miRNA-340-5p axis might act as a new ceRNA regulatory network to participate in the process of RCC. In addition, our study showed for the first time that silencing LINC00641 suppressed the proliferation and invasion abilities of RCC cells by targeting the LINC00641/miRNA-340-5p axis, which indicated that LINC00641 could be used as a potential therapeutic target for the precise treatment of RCC.

## Data Availability

The datasets from Zhongshan Hospital, Fudan University are available from the corresponding author on reasonable request.
